# Athlete’s Heart in Elite Biathlon, Nordic Cross—Country and Ski-Mountaineering Athletes: Cardiac Adaptions Determined Using Echocardiographic Data

**DOI:** 10.3390/jcdd9010008

**Published:** 2021-12-29

**Authors:** Paul Zimmermann, Othmar Moser, Max L. Eckstein, Jan Wüstenfeld, Volker Schöffl, Lukas Zimmermann, Martin Braun, Isabelle Schöffl

**Affiliations:** 1 Department of Cardiology, Klinikum Bamberg, 96049 Bamberg, Germany; martin.braun@sozialstiftung-bamberg.de; 2 Interdisciplinary Center of Sportsmedicine Bamberg, Klinikum Bamberg, 96049 Bamberg, Germany; volker.schoeffl@me.com (V.S.); l.zmann@gmx.de (L.Z.); isabelle.schoeffl@me.com (I.S.); 3 Division of Exercise Physiology and Metabolism, Department of Sport Science, University of Bayreuth, 95440 Bayreuth, Germany; othmar.moser@uni-bayreuth.de (O.M.); Max.Eckstein@unibayreuth.de (M.L.E.); 4 Interdisciplinary Metabolic Medicine Research Group, Division of Endocrinology and Diabetology, Medical University of Graz, 8036 Graz, Austria; 5 Insitute for Applied Exercise Science, University Leipzig, 04109 Leipzig, Germany; janwuestenfeld@hotmail.com; 6 Department of Traumatology and Orthopaedics, Klinikum Bamberg, 96049 Bamberg, Germany; 7 Department of Trauma and Orthopedic Surgery, FAU Erlangen-Nuremberg, 91054 Erlangen, Germany; 8 Section Wilderness Medicine, Department of Emergency Medicine, University of Colorado School of Medicine, Denver, CO 80045, USA; 9 School of Clinical and Applied Sciences, Leeds Becket University, Leeds LS1 3HE, UK; 10 Department of Pediatric Cardiology, University Hospital Erlangen-Nuremberg, 91054 Erlangen, Germany

**Keywords:** athlete’s heart, Biathlon, Nordic Cross—Country, Ski-mountaineering, cardiac remodeling, echocardiography

## Abstract

Twelve world elite Biathlon (Bia), ten Nordic Cross Country (NCC) and ten ski-mountaineering (Ski-Mo) athletes were evaluated for pronounced echocardiographic physiological cardiac remodeling as a primary aim of our descriptive preliminary report. In this context, sports-related cardiac remodeling was analyzed by performing two-dimensional echocardiography including speckle tracking analysis as left ventricular global longitudinal strain (LV-GLS). A multicenter retrospective analysis of echocardiographic data was performed in 32 elite world winter sports athletes, which were obtained between 2020 and 2021 during the annual medical examination. The matched data of the elite world winter sports athletes (14 women, 18 male athletes, age: 18–35 years) were compared for different echocardiographic parameters. Significant differences could be revealed for left ventricular systolic function (LV-EF, *p* = 0.0001), left ventricular mass index (LV Mass index, *p* = 0.0078), left atrial remodeling by left atrial volume index (LAVI, *p* = 0.0052), and LV-GLS (*p* = 0.0003) between the three professional winter sports disciplines. This report provides new evidence that resting measures of cardiac structure and function in elite winter sport professionals can identify sport specific remodeling of the left heart, against the background of training schedule and training frequency.

## 1. Introduction

Ski mountaineering (Ski-Mo) is one of the most endurant sports imaginable, as it involves the whole body, mostly performed at altitude involving uphill locomotion, representing a high-energy-demanding elite winter sport [1,2,3,4,5,6]. Biathlon (Bia) and Nordic Cross Country (NCC) are different to Ski-Mo, yet not less complex and metabolically demanding in their physiological demands, which have been of interest in previous studies [7,8]. The athletic features of these extreme endurance sports should cause pronounced structural and hemodynamical cardiac remodeling of the left heart [9,10,11]. As the training schedule as well as the conditions during training and competition are comparable between these three winter sports, they represent the athletes in our descriptive preliminary report, in which the most pronounced cardiac adaptations are to be expected.

The term athlete’s heart describes different changes and adaptions, namely structural, functional, physiological, and electro-physiological, due to sport-specific cardiac remodeling [12]. Different training stimuli (dynamic vs. static) lead to varying physiological adaptions [13,14]. In this context, especially pronounced changes due to left ventricular (LV) and atrial remodeling can be proven in elite endurance athletes [15]. Inherited and acquired cardiovascular abnormalities such as structural components as hypertrophic cardiomyopathies (HCM), arrhythmogenic ventricular cardiomyopathy (AVCM), or myocarditis—detectable via electrocardiographic (ECG) or echocardiographic evaluation—are sometimes difficult to be distinguished from the physiological adaptations observed in the so-called athlete’s heart, especially in athletes performing sports with a high dynamic component [16,17]. Therefore, regular standardized echocardiographic evaluation of athletes is essential, which might contribute to the prevention of undesirable cardiac events in young competitive athletes, because sudden cardiac death in athletes is one of the leading causes of mortality in athletes during sport activities [18,19,20,21,22,23,24,25].

In our preliminary report of elite winter sport athletes, we want to focus on the exercise-induced cardiac remodeling of the athlete’s heart by two-dimensional echocardiography including longitudinal peak strain examination [13]. This method, particularly the longitudinal Peak Systolic Strain (LPSS) allows an evaluation of early features of functional and morphological modifications in both ventricles, while conventional morphological echocardiographic parameters fail to distinguish the adaptations in the athlete’s heart [13,26,27,28,29,30,31,32,33]. In the last decade, strain analysis by speckle tracking echocardiography has emerged as an effective tool for sports cardiologists to assess the nature of hypertrophy and myocardial contractility especially in athletes [34]. Nevertheless, there is a lack of data about cardiac remodeling in world elite winter sports athletes. 

Therefore, the aim of the present descriptive preliminary report is to detect functional and morphological cardiac remodeling in extreme winter sports athlete’s heart.

## 2. Materials and Methods

The local ethics committee of the University of Nurnberg-Erlangen approved the study protocol (17_21 B). The study was conducted in conformity with Good Clinical Practice and the declaration of Helsinki [35]. Before any trial-related activities, our participants were informed about the study protocol and participants gave their written informed consent.

### 2.1. Study Population

**Figure 1:**
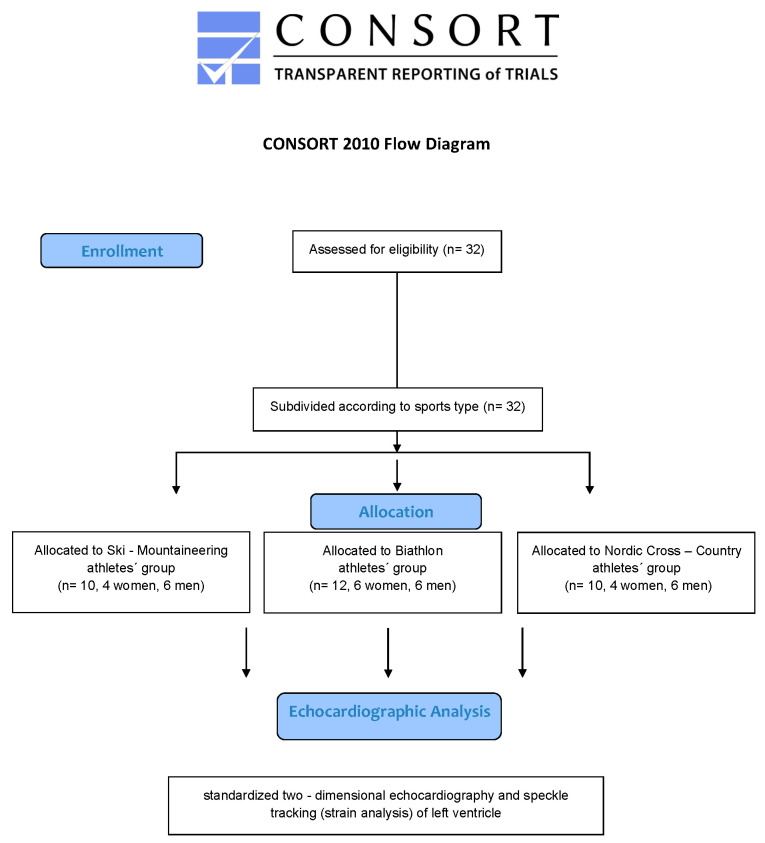
Flowchart of this study.

Thirty-two young elite winter sport professionals, all active members of the German National Team, participating in world cups and world championships, were examined during the season 2020/2021. During the severe COVID-19 pandemic situation, no participant was infected or had to be excluded from the analysis due to post-COVID-19 infection syndromes. All participants underwent a sports medicine check-up in their sport medicine performance center—Institute for Applied Exercise Science, University Leipzig and Interdisciplinary Center of Sportsmedicine, Klinikum Bamberg in the preseason preparation summer time. All participants were professional athletes with a total amount of 20–25 training hours per week in high-volume training time and 5–10 training hours in recreation time. Neither arrhythmias nor a known cardiac familiar defect have been detected during their sports career. Figure 1 shows the winter sport professionals selection flowchart.

### 2.2. Participants Visit

As part of the sports medicine checkup, we performed an echocardiographic functional and morphological assessment using a commercially available echocardiographic system Phillips EPIQ 7 device with an X5-1 aMatrix-array transducer (Phillips Healthcare, Eindhoven, The Netherlands), following a standard protocol [36]. The images were stored and analyzed digitally; for measurements, sequences of at least three heart beats were stored and analyzed. Participants were screened in the preseason preparation summer time and during the echocardiographic examination the heart rate and blood pressure were recorded for the following analysis. Two-dimensional echocardiographic analyses were performed following the general recommendations [36,37,38]. The systolic LV ejection fraction (LV-EF) was estimated and calculated using biplane Simpson rule, based on the apical four—as well as the apical two-chamber view. Two-dimensional linear dimensions for both ventricles and both atria were performed manually according to the recommendations [36,37,38]. An estimation of the right ventricular (RV) systolic function using the TAPSE (Tricuspide anular plane systolic excursion) was obtained in the apical four chamber view. 

Based on the two-dimensional echocardiographic measurements, the left ventricular mass index (LV Mass index) was calculated with a validated method [39], the relative wall thickness (RWT) of the left ventricle (LV) was calculated as (2× posterior wall thickness)/LVedd [39], and the left atrial volume index (LAVI) was obtained with a validated method for each participant [40]. 

For assessment of the LV diastolic function, we measured the pulse-wave Doppler in the apical four-chamber view referring to the peak early filling (E wave) and late diastolic filling (A wave) velocities. A tissue Doppler imaging of the lateral mitral anulus in the apical four chamber view was performed (peak early velocity E′) [36,37].

Furthermore, a LV speckle tracking analysis of the athlete’s heart was recorded, focusing on the LV-GLS pattern by two-dimensional strain in the apical views. We focused on the analysis of the LV and did not perform RV strain analysis.

Each of the participants was evaluated for the prevalence of right and left heart valve regurgitation as part of the standard echocardiographic assessment.

### 2.3. Statistical Analyses

Data were analyzed with Graph Pad Prism 8.2.1(279) (Graph Pad Software; San Diego, CA, USA) and Minitap statistic program (Minitab Inc.; State College, PA, USA). Our sample size was not normally distributed; therefore, we evaluated our numerical data group comparisons for ten Ski-Mo athletes, ten NCC athletes, and twelve elite Bia athletes using the analysis of variance testing (ANOVA) and nonparametric testing with post-hoc testing (*p* < 0.05). Afterwards, a gender-specific analysis for the interesting parameters was utilized equally.

## 3. Results

### 3.1. Baseline Characterictics and Echocardiographic Assessment

A total of 32 young professional winter sports athletes were examined. The matched data of the three different winter sports were compared for different echocardiographic parameters. In the two-dimensional echocardiographic examination, all participating elite winter sports athletes showed a low–normal to normal LV-EF estimated by the biplane Simpson method and did not show any relevant regurgitation of the left and right heart valves, except mild regurgitation at the mitral and tricuspid valve without any relevant systolic pulmonary artery pressure evaluated by tricuspid peak systolic velocity analysis. The heart rate at baseline as well as the blood pressure were quite low in this elite winter sport population. Anthropometric and echocardiographic data are shown in Table 1 and Table 2. 

Our measurement values were evaluated referring to the new standards and reference values in standardized transthoracic echocardiography as reported in the 2020 guidelines of the German Society of Cardiology (DGK) for untrained sedentary controls [38].

### 3.2. Morpholgical and Functional Cardiac Remodeling

**Figure 2:**
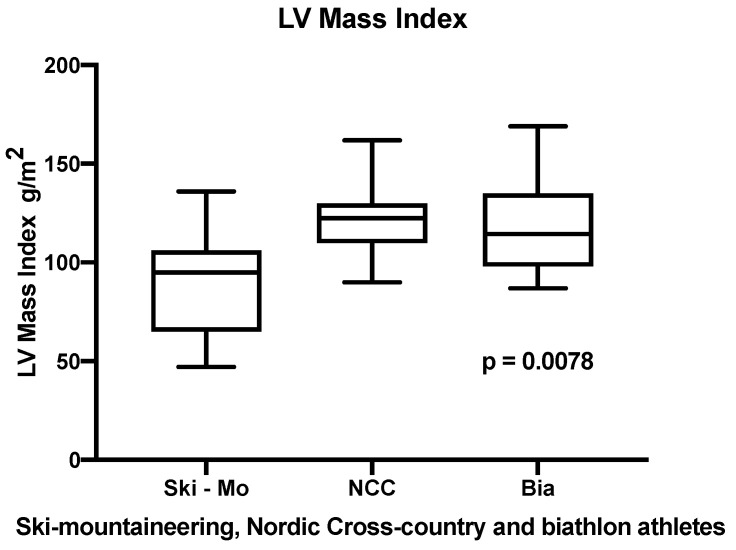
Left ventricular mass index significantly differs in elite winter sports athletes (*p* = 0.0078).

All athletes showed a normal systolic LV-EF, but the Bia athletes showed a significantly higher normal systolic LV-EF compared to Ski-Mo and NCC athletes (results shown in Table 2). In this context, analyzing the sex-specific differences of this parameter across the three disciplines, the female Bia-athletes showed a significantly higher LV-EF, compared to Ski-Mo and NCC athletes, whereby in our male participants no significant differences for LV-EF could be revealed (results shown in Table 2). The LV Mass index was calculated as an indexed parameter for all three different winter sports professionals and we could reveal significantly higher values for NCC and Bia athletes in comparison to Ski-Mo athletes (results shown in Table 2 and Figure 2). In this context, Bia and NCC athletes showed a significantly higher RWT in comparison to Ski-Mo athletes (results shown in Table 2).

**Figure 3:**
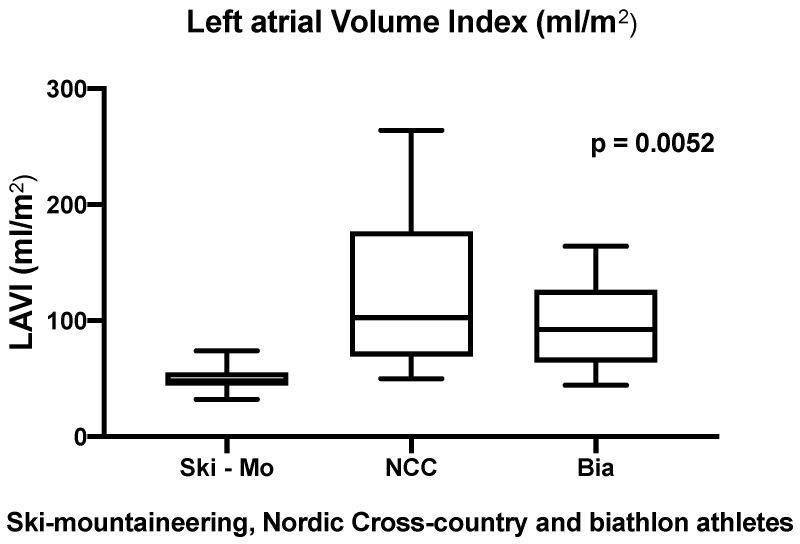
Analysis of the left atrial volume index (LAVI)—significant different results defined by the athletic sporting discipline (*p* *=* 0.0052).

Significant differences could be revealed in the echocardiographic analysis referring to LA remodeling of world elite athletes. The LAVI (mL/m^2^) was significantly enlarged in our NCC and Bia athletes in comparison to Ski-Mo athletes (results shown in Table 2 and Figure 3). Even sex-related differences between the three subdisciplines could be revealed for male NCC and Bia female athletes, which had significantly larger LAVI than the Ski-Mo athletes (results shown in Table 2).

The athletes differed significantly with regards to the eccentric remodeling of the LV, especially for the interventricular septal wall diameter (IVSd), the left ventricular posterior wall diameter (LVPWd) and RWT. NCC and Bia athletes showed thicker wall diameters than the Ski-Mo athletes, as shown in the Table 2. The gender-specific subanalysis revealed thicker LV wall diameter in the male and female NCC and Bia athletes than in the Ski-Mo athletes (results shown in Table 2). 

After proving sport-specific LA cardiac remodeling, the comparison of E/A and E/E′ ratio as criteria for LV diastolic function, revealed significant differences between NCC athletes and Bia athletes with respect to E/A, but not with respect to E/E′. The gender-specific subgroup analysis could not reach significant differences (results shown in Table 2).

No significant results could be revealed for the left ventricle end-diastolic size (LVedd), for the end-diastolic volume (LV EDVedd), the right heart dimensions as right atrial end-systolic diameter (RA endsyst) and right ventricular end-diastolic size (RV edd) as well as the TAPSE of RV in our three different elite winter sport disciplines. The sex-specific analysis across the three cohorts did not reach significant differences for the mentioned five parameters either, i.e., LVedd, LV EDVedd, RA endsyst, RV edd and TAPSE (LVedd and RA endsyst, results shown in Table 2).

**Figure 4:**
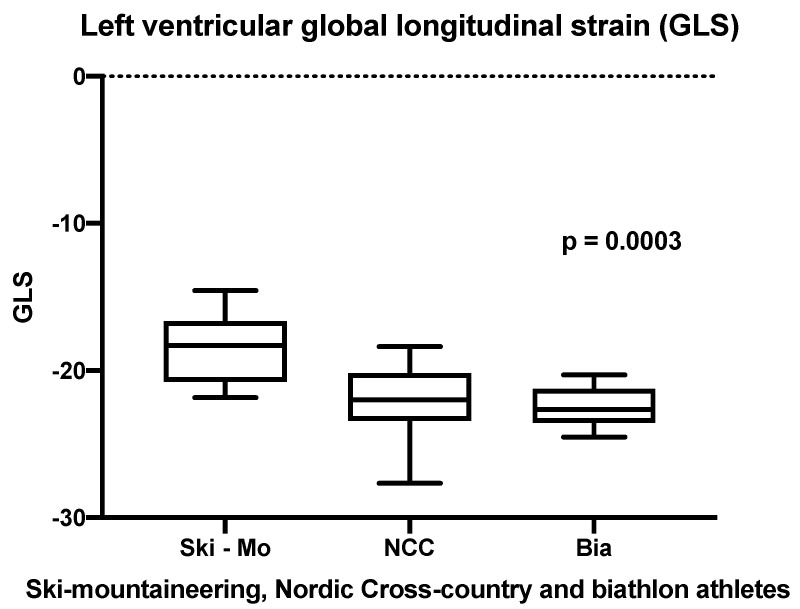
Analysis of the LV-GLS—significant differences defined by the athletic sporting discipline (*p* *=* 0.0003).

Focusing on the speckle tracking analysis with the main emphasis on the LV-GLS, significant differences defined by the athletic sporting discipline could be proven in our small cohort as represented in Figure 4, whereas the LV-GLS significantly differed in men with Ski-Mo athletes having the lowest values (*p* = 0.0003, shown in Table 2). 

Our measurements of the preliminary descriptive report were compared with the stated data of the German Society of Cardiology (DGK) position statement paper 2020 as generally sedentary control measurements, as presented in Table 3. Therefore, the LVedd diameter in our athletes is estimated to be normal for male participating athletes and in the upper normal range for female participants, and athletes’ LV-EF is estimated in the normal range for both. Our LV Mass index measurements, especially in the Bia and NCC athletes, have to be categorized in the upper normal to light extended range in comparison to the DGK sedentary controls. Remodeling of the LV, represented by IVSd and LVPWd, is classified in the upper normal to lightly thickened range in comparison to the DGK recommendations, especially in the Bia and NCC athletes.

## 4. Discussion

In the present descriptive preliminary report, the morphological and functional cardiac remodeling due to high-performance competition and training volume in elite winter sports athletes was investigated for the first time. The investigated winter sports in this study represent the most endurant competitive sports as they involve the whole body, are often performed at altitude and are associated with high and intense energy demands [1,2,3,7,8]. They combine ET and ST training components, and include mainly dynamic (isotonic) but also to a lesser degree static (isometric) elements [41].

### 4.1. Impact of Two Dimensional Echocardiography on Morpholgical and Functional Cardiac Remodeling of the Athlete’s Left Heart

The characteristics of the athlete’s heart have been investigated mostly in the LV as described by Utomi et al. [15]. The analysis of the systolic LV-EF in our elite winter sport professionals revealed, across the cohorts, significant differences of this weak parameter as seen for higher systolic LV-EF values for our Bia athletes. These results have to be handled carefully due to the fact that all athletes showed a normal systolic LV-EF and that other studies did not reveal significant differences between athletes and non-athletes [42,43]. Our Ski-Mo athletes showed the lowest, but still normal, systolic LV-EF parameters. 

Of the three disciplines, Ski-Mo represents the sport with the highest endurance component during training [2]. Most of their training regimen happens at or around ventilatory threshold 1 (VT1). The consequence is an associated bradycardia tendency and a low systolic cardiac function (LV-EF) at rest, when the heart works at a low capacity. In this context, the heart rate and blood pressure at baseline were quite low, as a common known phenomenon in elite athletes [23].

The LVedd and LV EDVedd did not reveal significant differences in our investigated groups of elite athletes, which showed a tendency for bradycardia in general, what has to be taken into consideration by evaluating this parameter. Another morphological remodeling key aspect is the induced LV hypertrophy in older athletes. Therefore, we evaluated in our cohorts the LV mass index, whereby Ski-Mo athletes showed significant lower values compared to NCC and Bia athletes as demonstrated in Table 2. 

These results must be handled carefully since the results may be due to inter-group differences in age, height and weight, BMI and BSA. Interindividual differences determined the group of Ski-Mo athletes as the physically smallest and youngest athletes, which might contribute to the obtained differences. Besides the physical status, the differences up to training schedules have to be taken into consideration. All participants in this study were professional athletes with a total amount of 20–25 training hours per week, but the Ski-Mo athletes were the youngest athlete category, with the fewest life time training hours and less years of participation as an elite athlete. The main training focus in Ski-Mo athletes is set on ET sessions, whereas ST sessions represent an essential part in Bia and NCC athletes’ training schedule. These circumstances have to be taken into consideration while investigating the progress of exercise-induced cardiac remodeling. Generally, a greater wall thickness of the LV is observed in athletes focusing on ST, i.e., static aspects [44]. In our cohort, these athletes are represented by the NCC and Bia athletes with greater wall thickness than Ski-Mo athletes, while the Ski-Mo athletes show a tendency for eccentric left ventricular wall remodeling due to the higher dynamic aspect of their training. These results—especially proportionately larger LV walls—were firstly proposed and described by Morganroth, but represent a quite common finding, even in our athletes [45]. These variable circumstances have to be taken into consideration once again, while evaluating sport-specific cardiac remodeling, mostly influenced by different aspects due to th the athlete’s constitution and different training variables. Nevertheless, our analyzed participants number is small, due to the fact that we only investigated athletes from the German national teams competing at world-class events. Our study has a rather small sample size which is restricted due to the limited spots on a national team. Consequently, drawing a conclusion or reflecting our results for the general population being involved in Ski-Mo, Bia or NCC would be misleading. Furthermore, as mentioned in the limitations section of our manuscript, an interobserver variability with respect to measurement acquirement must be considered.

Sports-specific remodeling consisted of left heart dimension adaption [46], in a concentric remodeling of the LV. This emerging concentric hypertrophy might be an adaption to sports-specific dynamic efforts in winter sports, but may also be a marker of a beginning heart disease and a regular routine analysis might contribute to prevention of sudden cardiac death in athletes. Our descriptive reporting of significant differences could be proven for LV remodeling, represented as following by RWT and data of LV wall diameters. These trends in data analysis have to be handled carefully due to the mentioned aspects of small size, variable training schedule and physical differences as proven by BMI and BSA inter-athlete differences. Nevertheless, these results show interesting tendencies, which should be confirmed in future studies with a greater number of participants.

In this controversy of training-related cardiac adaption and functional remodeling versus a beginning balanced cardiomyopathy in male endurance athletes, new insights were provided by Utomi et al. [15]. The authors noted morphological features, above all left heart remodeling, with enlarged left heart structures in male athletes in general. 

In our study, LA remodeling was observed especially in NCC and Bia athletes. Our evaluated parameter, LAVI, revealed in our small number of participants significantly different results, which implies significantly higher values for the NCC and Bia athletes compared to Ski-Mo athletes. These athletes are represented mainly as a homogenously young and physically small group of male and women athletes. Therefore, our results have to handled with care and can only serve as a description of structural cardiac remodeling of world professional winter sport athletes. Nevertheless, LA remodeling has been described as a typical characteristic in endurance athletes [46]. However, this common finding in highly trained endurance athletes might contribute to the development of an atrial cardiomyopathy, which is characterized by LA enlargement and fibrosis. There is some evidence that an accumulation of lifetime training hours and participation in competitions plays an important role for this development [25,47]. In this context, our results have to handled with care due to the clinical presentation of our athletes, which have even an increased risk for developing an atrial cardiomyopathy during their career and afterwards. These findings might also suggest an atrial remodeling due to firstly structural LAVI as well as secondly to functional remodeling, represented as LA passive and total emptying fraction. Kasikcioglu et al. revealed positive correlations between VO _2max_ and LA passive emptying fraction, which might contribute to improved exercise capacity in athletes and improved cardiac output during exercise [48]. On the other hand, Klasnja et al. found a mild association between measurement of resting left heart cardiac structure and function and peak cardiac output performance, whereas the interpretation of the association should be considered carefully, even in our presented data [49].

The functional remodeling of the LV can be evaluated using the peak early to late diastolic filling ration (E/A ratio) and tissue doppler analysis (E/E′ratio). Higher values are observed in athletes in comparison to non-athletes [50]. We found similar results in our study, which is comparable to previously published data [15]. However, functional remodeling is a controversially discussed topic, as the enhanced maximal cardiac output could be a consequence of enhanced diastolic function on the one hand but is also influenced by putative mechanisms such as volume preload or intrinsic relaxation conditions on the other hand [51]. Our descriptive results emphasize the training-induced adaptations in athletes, which support the thesis of a balanced cardiomyopathy that is supposed to be physiological, but has to be judged with respect to the specific sport and the lifetime training hours. A higher diastolic capacity of the LV might be explained by lower heart rates at rest, improved hemodynamic effects and an increased vagal component in athletes [50].

Nevertheless, discussing the specific cardiac remodeling of the winter sport elite athlete’s heart, it has to be stated clearly that the prognostic value of reported echocardiographic assessment is limited and does not provide sensitive results for at least two frequent conditions of sudden cardiac death in athlete’s heart. Firstly, coronary artery anomalies and secondly the non-compaction cardiomyopathy of the LV cannot be sensitively detected or ruled out by two-dimensional echocardiographic assessment. Therefore, further cardiac imaging techniques, such as computer tomography or cardiac MRI should be considered [52,53,54]. In this context, the cardiac MRI screening of Angelini et al. could reveal 1.3% of young people to have high risk cardiovascular conditions, with a surprisingly high value of unknown LV non-compaction cardiomyopathy in this cohort of adolescents. These MRI-based screening protocols might pave the road to accurately identify potential deadly cardiac abnormalities and prevent sudden cardiac death [52].

### 4.2. Global Longitudinal Strain (GLS) Analysis of the Athlete’s Left Ventricle in Winter Sport Professionals

Echocardiographic two-dimensional speckle tracking is a modern method for identifying subtle differences in the adaption of LV strain cardiac pattern or twist mechanism of the LV in athletes [27]. The distinction between physiological adaption and inherited or acquired HCM can be improved by using LV-GLS. In this context, normal LV-GLS is reported between −18% and −25% in healthy individuals, whereas strain imaging in general can reveal early changes and functional abnormalities in cardiac mechanics long before structural damages can be detected [55,56,57]. In our study limited by the small number of participating athletes, LV-GLS was slightly reduced in Ski-Mo—when compared to NCC and Bia athletes. On the one hand, as mentioned above, Ski-Mo athletes are represented mainly as a homogenously young and physically small group of male and female athletes with less life time training hours compared to the career of the athletes in NCC and Bia. It must be mentioned that our measurements are in the normal range of healthy subjects and were adapted in the preseason preparation summer time. Further sport-season specific echocardiographic follow-ups might be an interesting scientific research topic to evaluate the reversal of echocardiographic strain rate in the context of training intensity and training-related cardiac volume load. An interobserver variability might also finally contribute to our reported slightly existing differences between the different winter sport professionals. Nevertheless, our data confirm previous findings in which highly trained Olympic athletes showed normal GLS and strain rate parameters of the LV with merely mild differences compared with untrained controls [28]. On the other hand, different patterns of LV deformation mechanics were revealed early in young footballers [11,29]. In the end, subtle physiological differences of LV strain pattern analysis have to be interpreted with respect to the performed sport, as reported by Beaumont et al. [27]. In the end, our results emphasize the training-induced adaptions in athlete’s heart and although an atrial cardiomyopathy can not be stated clearly, there is evidence for an increased risk for degeneration from a balanced physiological cardiomyopathy to a pathological entity within the lifetime of a sports career. In this context, additional information might be provided by detection of atrial fibrosis in the cardiac MRI. 

### 4.3. Impact of Two Dimensional Echocardiography on Morpholgical and Functional Cardiac Remodeling of the Athlete’s Right Heart

The right side of the heart in athletes has also received some interest in the past as well as in the current scientific view. In a systematic review in athletes, Ascenzi et al. [58] detected training-induced RV enlargement. However, RV dilatation is a common phenotypic expression and a differential diagnosis for ARVC. Therefore, the sports-specific background needs to be taken into account in order to discriminate physiological adaption from cardiomyopathies. As a limitation, we did not perform RV strain analysis in our study, nevertheless this topic represents an interesting tool for functional right heart remodeling. In this context, an improved RV apical strain in comparison to a decrease of RV strain in the basal segments is described in athletes in various studies. The specific regional differences within the RV are highlighted, with a broad variability among athletes and in terms of training load [59,60,61]. Morphological right heart adaption in elite endurance athletes is a common finding in several studies [50], and is reported to show greater right heart remodeling than in ST athletes and untrained controls [62]. Our elite winter sport athletes, which are characterized by ET as a major component as well as speed- and ST units [63], showed comparable results with age- and sex-matched normal data when investigating the right heart echocardiographic parameters (RA and RV size, TAPSE). There was no significant difference between our three elite winter sport professional athlete’s groups. However, changes in the right heart are reported to be small and do not prohibit peak performance [50]. Regarding all these different morphological parameters and influencing circumstancing facts, due to the small number of participants, different anatomical athletes’ structure and training schedules, our findings can provide guidance for further investigation with a greater number of participating athletes.

### 4.4. Limitations

Our study has several limitations as mentioned above in the discussion. First of all, the number of professional winter sport athletes is relatively small which is due to the fact that we only investigated high-level athletes from the German national teams competing at world-class events. Secondly, Ski-Mo athletes were the youngest athlete category, implying fewer lifetime training hours and showed a smaller physical constitution than NCC and Bia athletes—these circumstances have to be taken into consideration, while evaluating sport-specific cardiac remodeling and we agree that our paper should be likely regarded as an interesting descriptive preliminary report. Furthermore, the echocardiographic analyses of the participating athletes were performed in a multicenter study design, so that an interobserver variability with respect to measurement acquirement must be considered on the one hand. On the other hand, the mixture of young and experienced athletes especially in the NCC and Bia athlete cohort entails an interindividual variability in relation to anatomical habitus, lifetime training hours and training schedule variability, which might contribute to a certain standard deviation in our measurements. Thirdly, the multicenter measurement was performed in the preseason preparation time in summer. This condition associated with individual training schedules might contribute to individual athlete’s heart volume change and result in this difference. Our functional left ventricular analysis has been focused on the LV-GLS and not on the circumferential strain analysis of the LV. No specific strain analysis has been performed in the RV, which is an important limitation of this study and might be performed in further investigations.

## 5. Conclusions

This is the first study to present data of world class winter sports athletes and morphological and functional remodeling of the left heart.

Our results have to be handled with care due to the mentioned limitations and serve as a preliminary report. Therefore, our results analysis—in general as well as in the gender-specific subgroup—can identify physiological differences in functional and morphological cardiac remodeling, especially due to LV-EF and LV mass index, LA remodeling as measured by LAVI, and differences in the speckle tracking analysis, focusing the LV-GLS. Nevertheless, the individual morphological and secondary functional adaption of the athlete’s heart have to be interpreted carefully due to the different types of sports and lifetime training hours. From this aspect, future studies should consider a greater number of participating athletes to verify the impact on sport-specific cardiac remodeling and to further strengthen the evidence base.

## Figures and Tables

**Table 1:** Baseline winter sport professional characteristics. Anthropometric data of the Ski-Mo, NCC and Biathlon athletes.

	Ski–Mo *n* = 10		NCC *n* = 10		Biathletes *n* = 12	
	Male *n* = 6	Female *n* = 4	Male *n* = 6	Female *n* = 4	Male *n* = 6	Female *n* = 6
Age (y)	21.2 ± 1.9	20.8 ± 2.4	26.3 ± 4.1	25.5 ± 0.5	27.3 ± 3.6	29.0 ± 3.2
Height (cm)	178.4 ± 3.7	163.5 ± 8.8	181.3 ± 4.7	171.2 ± 5.8	180.9 ± 5.1	172.8 ± 3.7
Weight (kg)	67.5 ± 0.5	53.2 ± 6.5	72.0 ± 3.0	63.4 ± 5.9	77.1 ± 3.7	62.5 ± 4.1
BMI (kg/m^2^)	18.9 ± 1.7	19.8 ± 0.4	22.0 ± 1.1	21.6 ± 1.2	23.6 ± 0.9	20.9 ± 1.0
Resting Blood Pressure Systolic/Diastolic (mmHg)	120 ± 5.6 82 ± 3.5	100 ± 8.2 72 ± 1.5	125 ± 8.3 78 ± 2.9	105 ± 7.2 71 ± 3.8	117 ± 7.6 77 ± 2.2	108 ± 6.2 70 ± 3.3
Resting Heart Rate (bpm)	40 ± 5.6	44 ± 4.5	42 ± 3.6	46 ± 5.1	41 ± 4.2	45 ± 5.1
BSA (body surface area m^2^)	1.75 ± 0.08	1.61 ± 0.12	1.88 ± 0.04	1.81 ±0.07	1.92 ± 0.04	1.77 ± 0.05

Data are presented as a median with standard deviation.

**Table 2:** Echocardiographic measurements (mean ± SD) of the Ski-mountaineering, Nordic Cross—Country and Biathlon athletes.

	Ski-Mo (I) *n* = 10		NCC (II) *n* = 10		Biathletes (III) *n* = 12		*p*-Value
	Male *n* = 6	Female *n* = 4	Male *n* = 6	Female *n* = 4	Male *n* = 6	Female *n* = 6	
LV edd (mm)	50.83 ± 4.22	45.25 ± 5.96	55.50 ± 3.83	50.75 ± 3.50	55.50 ± 5.24	49.50 ± 1.52	ns
	48.6 ± 5.48		53.6 ± 4.27		52.5 ± 4.83		
LV Mass Index (g/m^2^)	97.2 ± 25.2	76.3 ± 26.7	130.7 ± 16.5	106 ± 16.4	133.5 ± 20.6	102.3 ± 14.8	0.0078 *
	88.8 ± 26.6 *		120.8 ± 20.1 *		117.9 ± 23.6 *		
Relative Wall Thickness RWT	0.38 ± 0.03	0.34 ± 0.06	0.40 ± 0.04	0.41 ± 0.04	0.40 ± 0.04	0.42 ± 0.04	Ski-Mo vs. NCC 0.0230 * Ski-Mo vs. Bia 0.0230 *
	0.37 ± 0.05		0.41 ± 0.03		0.41 ± 0.04		
IVSd (mm)	8.67 ± 1.97	8.25 ± 2.50	11.00 ± 0.63	10.50 ± 0.58	10.83 ± 0.98	9.67 ± 1.37	Ski-Mo vs. NCC 0.0266 *, Ski-Mo vs. Bia 0.0337 *
	8.5 ± 2.07		10.4 ± 1.17		10.3 ± 1.29		
LVPWs (mm)	9.97 ± 1.03	7.75 ± 1.50	11.17 ± 0.41	10.50 ± 0.58	12.33 ± 2.07	10.17 ± 1.17	Ski-Mo vs. NCC 0.0161 * Ski-Mo vs. Bia 0.0030 *
	8.9 ± 1.52		10.9 ± 0.57		11.3 ± 1.96		
E/A	2.18 ± 0.58	1.98 ± 0.17	2.48 ± 0.26	2.40 ± 0.77	1.97 ± 0.52	1.75 ± 0.40	NCC vs. Bia 0.0166 *
	2.1 ± 0.45		2.5 ± 0.49		1.9 ± 0.46		
E/E′	6.75 ± 1.71	7 ± 1.79	6.80 ± 0.86	6.13 ± 1.22	7 ± 0.86	6.37 ± 1.04	ns
	6.9 ± 1.66		6.4 ± 1.09		6.7 ± 0.97		
LAVI (mL/m^2^)	51.83 ± 12.1	46.25 ± 11.1	150 ± 84.58	89.3 ± 45.7	117.5 ± 37.7	72.8 ± 19.6	0.0052 *
	49.6 ± 11.4		125.7 ± 75.2		95.2 ± 36.9		
RA (cm^2^)	19.17 ± 3.87	16.75 ± 2.87	24.83 ± 3.73	18.28 ± 4.72	20.78 ± 3.64	15.50 ± 2.40	ns
	18.2 ± 3.55		22.2 ± 5.16		18.1 ± 4.03		
LV–EF	60,33 ± 4.08	58.00 ± 5.60	61.17 ± 4.96	59.50 ± 4.80	65.83 ± 5.38	73.00 ± 4.34	0.0001 *
	59.4 ± 4.60		60.5 ± 4.70		69.4 ± 5.98		
GLS	−18.26 ± 2.21	−18.83 ± 2.93	−21.21 ± 1.99	−23.25 ± 3.23	−22.62 ± 1.26	−22.34 ± 1.42	0.0003 *
	−18.5 ± 2.38		−22.0 ± 2.61		−22.5±1.29		

Data are presented as mean with standard deviation. *p* value *, statistically significant (*p* < 0.05). Abbreviations: LV edd, left ventricle enddiastolic size; LV, left ventricular; IVSd, interventricular septal wall thickness at diastole; LVPWd, left ventricular posterior wall thickness at diastole; E/A and E/E, parameters for diastolic function of the left ventricle; LAVI, left atrial volume index; RA, right atrium; LV-EF, left ventricular systolic ejection fraction; GLS, global longitudinal strain; ns, non-significant.

**Table 3:** Comparison of our structural two dimensional echocardiographic data to the stated control data of the position statement paper of DGK (German Society of Cardiology, 2020).

Parameter	OUR Study Data		Reference Value Male	Reference Value Female
	Total Male (*n* = 18)	Total Female (*n* = 14)		
LV edd (mm)	53.9 ± 4.8	48.6 ± 4.1	42–58	38–52
LV-EF (%)	62.4 ± 5.2	64.9 ± 8.6	52–72	54–74
LV Mass Index (g/m^2^)	120.4 ± 26.1	95.9 ± 21.9	49–115	43–95
IVSd (mm)	10.2 ± 1.7	9.2 ± 1.7	6–10	6–9
LVPWd (mm)	11.1 ± 1.7	9.6 ± 1.6	6–10	6–9

Data are presented as mean with standard deviation. LV edd, left ventricular enddiastolic size; LV-EF, left ventricular systolic ejection fraction; LV, left ventricular; IVSd, interventricular septal wall thickness at diastole; LVPWd, left ventricular posterior wall thickness at diastole; mm, millimeter; g, grams; m^2^, square meters.

## Data Availability

Individual anonymized data supporting the analyses of this study contained in this manuscript will be made available upon reasonable written request from researchers whose proposed use of data for a specific purpose has been approved.

## Abbreviations

Bia = Biathletes, NCC = Nordic Cross—Country, Ski-Mo = Ski mountaineering, GLS = global longitudinal strain, HCM = hypertrophic cardiomyopathies, AVCM = arrhythmogenic ventricular cardiomyopathy, LV = left ventricle, LV-EF = left ventricular systolic function, LV edd = left ventricular enddiastolic diameter, BSA = body surface area, LV Mass Index = left ventricular mass index, RA = right atrium, LA = left atrium, LAVI = left atrial volume index, RV = right ventricular, TAPSE = Tricuspide anular plane systolic excursion, RWT = relative wall thickness, ANOVA = analysis of variance testing, BMI = Body mass index, IVSd = interventricular septal wall thickness at diastole, LVPWd = left ventricular posterior wall thickness at diastole, E/A and E/E′= parameters for diastolic function of the left ventricle, DGK = German Society of Cardiology, VT1 = ventilatory threshold 1, ET = endurance training, ST = strength training.
